# Categorization of the Ocular Microbiome in Japanese Stevens–Johnson Syndrome Patients With Severe Ocular Complications

**DOI:** 10.3389/fcimb.2021.741654

**Published:** 2021-11-19

**Authors:** Mayumi Ueta, Koji Hosomi, Jonguk Park, Kenji Mizuguchi, Chie Sotozono, Shigeru Kinoshita, Jun Kunisawa

**Affiliations:** ^1^ Department of Ophthalmology, Kyoto Prefectural University of Medicine, Kyoto, Japan; ^2^ Laboratory of Vaccine Materials, Center for Vaccine and Adjuvant Research and Laboratory of Gut Environmental System, National Institutes of Biomedical Innovation, Health and Nutrition (NIBIOHN), Ibaraki, Japan; ^3^ Laboratory of Bioinformatics, Artificial Intelligence Center for Health and Biomedical Research, National Institutes of Biomedical Innovation, Health and Nutrition (NIBIOHN), Ibaraki, Japan; ^4^ Institute for Protein Research, Osaka University, Suita, Japan; ^5^ International Research and Development Center for Mucosal Vaccines, The Institute of Medical Science, The University of Tokyo, Tokyo, Japan; ^6^ Graduate School of Medicine, Graduate School of Dentistry, Osaka University, Suita, Japan; ^7^ Department of Microbiology and Immunology, Kobe University Graduate School of Medicine, Kobe, Japan

**Keywords:** Stevens–Johnson syndrome (SJS), ocular microbiome, *Corynebacterium*, Neisseriaceae, *Staphylococcus*, mucosal immunity, inflammation, toxic epidermal necrolysis (TEN)

## Abstract

The commensal microbiota is involved in a variety of diseases. Our group has noticed that patients with Stevens–Johnson syndrome (SJS)/toxic epidermal necrolysis (TEN) often present with persistent inflammation of the ocular surface, even in the chronic stage, and that this inflammation is exacerbated by colonization of the mucosa by certain bacteria. However, the changes in the composition of the ocular microbiome in SJS/TEN patients with severe ocular complications (SOCs) remain to be fully investigated. Here, we conducted a cross-sectional study of 46 Japanese subjects comprising 9 healthy control subjects and 37 SJS/TEN patients with SOC. The 16S rRNA-based genetic analyses revealed that the diversity of the ocular microbiome was reduced in SJS/TEN patients with SOC compared with that in healthy control subjects. Principal coordinate analysis based on Bray–Curtis distance at the genus level revealed that the relative composition of the ocular microbiome was different in healthy control subjects and SJS/TEN patients with SOC, and that the SJS/TEN patients with SOC could be divided into four groups based on whether their microbiome was characterized by enrichment of species in genus *Corynebacterium 1*, *Neisseriaceae uncultured*, or *Staphylococcus* or by simultaneous enrichment in species in genera *Propionibacterium*, *Streptococcus*, *Fusobacterium*, *Lawsonella*, and *Serratia*. Collectively, our findings indicate that enrichment of certain bacteria at the ocular surface could be associated with ocular surface inflammation in SJS/TEN patients with SOC.

## Introduction

Stevens–Johnson syndrome (SJS) is an acute inflammatory vesiculobullous reaction of the skin and mucosae, including those of the eyes, oral cavity, and genitals. In patients with extensive skin detachment and a poor prognosis, the condition is called toxic epidermal necrolysis (TEN). Together, SJS and TEN form a spectrum of disease, with SJS being less severe and TEN being more severe.

Ophthalmic symptoms of SJS/TEN occur at the ocular surface and can often lead to visual loss, although only about half of all cases of SJS/TEN develop severe ocular lesions. In the acute stage, these lesions can include severe pseudomembranous conjunctivitis and ocular surface epithelial defects (Sotozono et al., 2015), which are followed by severe ocular sequelae such as dry eye, trichiasis, symblepharon, and conjunctival invasion into the cornea, which negatively affect patients’ quality of life (Sotozono et al., 2007).

When patients present in the chronic stage, it is difficult for ophthalmologists to render a differential diagnosis of either SJS or TEN because the vesiculobullous skin lesions expressed in the acute stage have healed by the chronic stage (Ueta and Kinoshita, 2012). Diagnosis of ocular SJS/TEN is usually based on a confirmed history of acute-onset high fever, serious mucocutaneous illness with skin eruptions, and involvement of at least two mucosal sites including the ocular surface (Ueta et al., 2007b; Ueta et al., 2007c; Ueta et al., 2014a; Ueta et al., 2014b; Ueta et al., 2015), as well as the presence of severe ocular sequelae. Our group defines SJS/TEN patients with severe ocular complications (SOCs) as those in the acute stage presenting with severe pseudomembranous conjunctivitis and epithelial defects on the ocular surface (cornea and/or conjunctiva) (Sotozono et al., 2009), and those in the chronic stage as presenting with severe ocular sequelae. Ophthalmologists tend to report both SJS and TEN with SOC as “SJS” in a broad sense (Ueta and Kinoshita, 2012).

Our group has noticed that SJS/TEN patients with SOC tend to present with opportunistic bacterial infection of the ocular surface, particularly by methicillin-resistant strains of *Staphylococcus aureus* and *S. epidermidis* (MRSA/MRSE). Indeed, we previously reported that the detection rate of MRSA and MRSE on the ocular surface of SJS/TEN with SOC patients was higher compared with that in individuals with other major ocular surface disorders (Sotozono et al., 2002). We have also noticed that SJS/TEN patients with SOC tend to present with persistent inflammation of the ocular surface, even in the chronic stage, and that this ocular surface inflammation is exacerbated by colonization by MRSA/MRSE; in healthy individuals, colonization by these bacteria would likely not induce ocular surface inflammation (Ueta and Kinoshita, 2012). Thus, we suspect there is an association between SJS/TEN with SOC and a disordered mucosal innate immunity (Ueta and Kinoshita, 2012). To examine this hypothesis further, we examined the ocular surface microbiome of SJS/TEN patients with SOC and compared it with that of healthy controls.

## Materials and Methods

### Patients and Controls

Thirty-seven Japanese SJS/TEN patients with SOC (22 females, 15 males; age range 17 to 81 years; mean age, 52.19 ± 16.90) were independently recruited at the Kyoto Prefectural University of Medicine. The diagnosis of SJS/TEN with SOC was based on a confirmed history of acute-onset high fever, serious mucocutaneous illness with skin eruptions, and the involvement of at least two mucosal sites including the oral cavity and ocular surface, and the presence of severe ocular sequelae. Ten patients had contact lenses (six hard, four soft) for care of their ocular surface. All patients used some antibiotic eye drops for their increasing discharge. Nine healthy Japanese volunteers (five females, four males; age range 17 to 81 years; mean age 43.33 ± 24.82) were also recruited. All experiments were approved by both the committees of the National Institutes of Biomedical Innovation, Health, and Nutrition and Kyoto Prefectural University of Medicine and were conducted in accordance with their guidelines. Informed consent was obtained from all participants. We collected discharge from both eyes of all subjects, and when amplification by PCR was successful from discharge of both eyes, the right eye was selected in comparison between SJS/TEN with SOC and controls. Sterile cotton swabs were used, and discharges were carefully collected from the lower conjunctival sac to avoid contamination.

### 16S rRNA Gene Amplicon Sequencing Analysis

DNA extraction from human ocular samples was performed using Quick Gene DNA tissue kit S and Quick Gene-Mini80 machine (Kurabo Industries Ltd., Osaka, Japan) in accordance with the manufacturer’s instructions. 16S rRNA gene amplicon sequencing was performed with DNA from human ocular microbiome by modifying our methods for fecal bacteria (Hosomi et al., 2017). The V3–V4 region of the 16S rRNA gene was amplified from the ocular DNA samples using the following primers: forward, 5-TCGTCGGCAGCGTCAGATGTGTATAAGCGACAGCCTACGGGNGGCWGCAG-3, and reverse, 5-GTCTCGTGGGCTCGGAGATGTGTATAAGAGACAGGACTACHVGGGTATCTAATCC-3 (Klindworth et al., 2013). Reactions were carried out in 20 µl solutions containing 0.2 mM dNTPs, 0.6 µM of each primer, 0.5 U Takara Taq (Takara Bio Inc., Shiga, Japan), 1 × PCR buffer, and sample DNA. The following thermal cycling conditions were used: initial denaturation at 95°C for 3 min, followed by 40 cycles consisting of denaturation (95°C for 30 s), annealing (55°C for 30 s), and extension (72°C for 30 s), and a final extension step at 72°C for 5 min. Amplicons were purified using the AMPure XP (Beckman Coulter, Inc., CA, USA), a DNA library was prepared by using a Nextera XT Index kit (Illumina Inc., CA, USA), and 16S rRNA gene sequencing was performed by using a MiSeq system (Illumina). The sequencing results were analyzed by using the Quantitative Insights Into Microbial Ecology (QIIME) software package v1.9.1 (Caporaso et al., 2010) and QIIME Analysis Automating Script (Auto-q) (doi: 10.5281/zenodo.1439555) as previously described (Mohsen et al., 2019). Open-reference OTU picking and taxonomy classification were performed based on sequence similarity (>97%) using UCLUST software v6.1 (Edgar, 2010) with the SILVA v128 reference sequence (Quast et al., 2013). Ten thousand reads per sample were randomly selected for further analysis. Samples with insufficient read numbers were re-sequenced, and samples with repeated insufficient read numbers were thereafter excluded. To avoid the detection of contaminants, DNA extracted from unused sterile cotton swabs was analyzed as a negative control. We confirmed that 16S rRNA gene was not amplified, and less than 223 reads per sample were obtained from the sequencing of triplicate negative control samples including genera not typical in the ocular samples such as *Bacteroides* and *Massilia* (**Table S1**). These findings collectively indicate the little contaminants from the cotton swab.

### Bioinformatic Analysis

The output of the QIIME pipeline, in the BIOM file format, was imported into the R software environment (version 3.5.1) for analysis. Alpha-diversity indices were calculated by using the estimate_richness function in the “phyloseq” R package. Beta-diversity index, calculated by using Bray–Curtis distance and genus-level data, was determined by using the vegdist function in the “vegan” R package. Principal coordinate analysis was performed by using the dudi.pco function in the “ade4” R package. Hierarchical clustering analysis was performed using the “vegan” and “stats” R packages, based on Bray–Curtis distance matrix at the genus level, and by using the ward.D2 method (Murtagh and Legendre, 2014). Principal coordinate analysis figures were created by using the “ggplot2” R package. BlastN searches of representative OTU sequences were conducted using the rRNA_typestrains/16S_ribosomal_RNA database (last updated 2021/02/16) (Zhang et al., 2000). Statistical significance was evaluated by one-way ANOVA for comparison of multiple groups and Mann–Whitney *U*-test or Wilcoxon rank sum test for two groups by using Prism 7 software (GraphPad Software, CA, USA).

### Bacterial Culture Test

Potential bacterial pathogens in the eye sebum of the conjunctival fornix were identified by broth-enrichment culture methods. Samples were swabbed from the patient’s conjunctiva, mixed with 2 ml of broth, and incubated overnight at 35°C. One μl of broth was plated onto blood agar and chocolate agar and further incubated overnight at 37°C with 5% CO_2_. Potential bacteria were then identified by matrix-assisted laser desorption ionization–time of flight mass spectrometry (MALDI-TOF MS) using MALDI Biotyper (Bruker Daltonik GmbH, Bremen, Germany).

## Results

### Reduced Ocular Bacterial Diversity in SJS/TEN Patients With SOC

**Figure 1:**
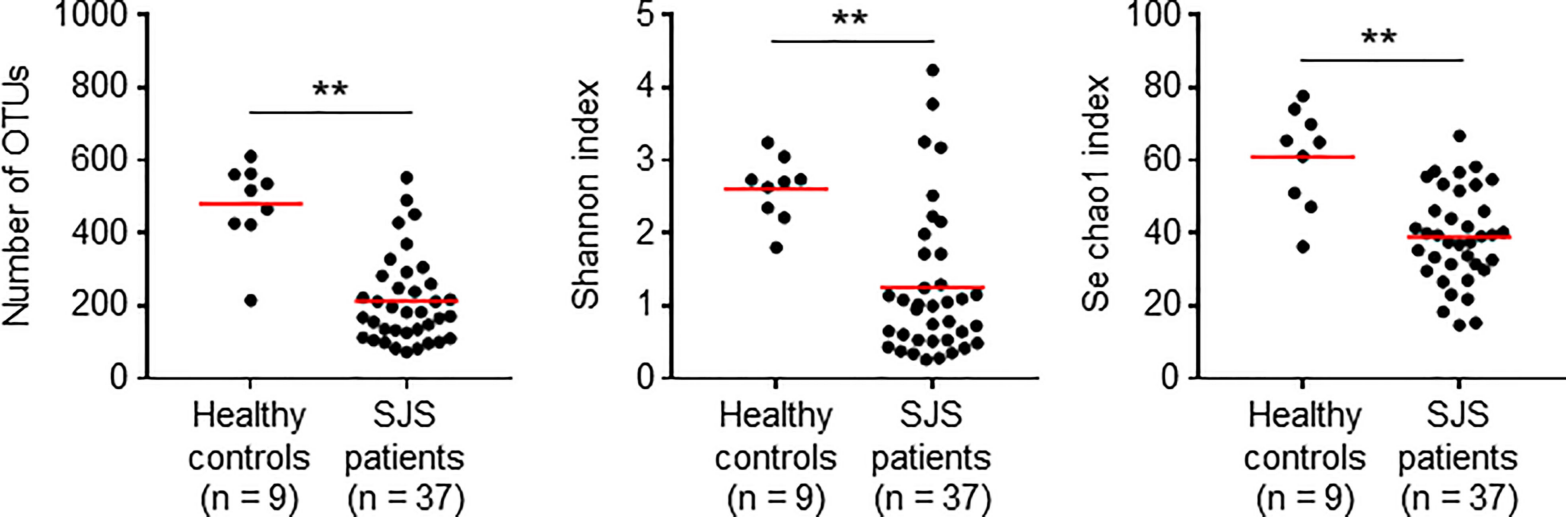
Reduced alpha diversity in SJS/TEN patients with SOC. Statistical significance was evaluated by using the Mann–Whitney *U*-test; **p < 0.01. OTU, operational taxonomic unit. SJS, SJS/TEN patients with SOC.

To assess the association between the ocular microbiome and SJS/TEN patients with SOC, we conducted a cross-sectional study of 46 Japanese participants comprising 9 healthy control (HC) subjects and 37 SJS/TEN patients with SOC. The age and sex of each subject are shown in **Table 1**. The number of operational taxonomic units (OTUs) identified was significantly reduced in SJS/TEN patients with SOC compared with that in HC subjects (**Figure 1**). In addition, the Shannon and Se chao1 indexes, which express evenness and richness in bacterial diversity, respectively, were also significantly reduced in SJS/TEN patients with SOC. Actually, 22 of 37 (59.5%) SJS/TEN patients with SOC show their first population bacteria account for more than 90% of the relative abundance (**Table 1B**), but none of HC subjects (**Table 1A**). In contrast, 6 of 9 (66.7%) HC subjects show their first population bacteria account for less than 70% of the relative abundance (**Table 1A**), but 8 of 37 (21.6%) SJS/TEN patients with SOC (**Table 1B**). Thus, although it may be due to the fact that the patients are receiving antibiotics, these results suggest there is reduced bacterial diversity on the ocular surface of SJS/TEN patients with SOC compared with that in HC subjects.

**Table 1A:** Predominant genera in healthy controls.

Age			Sex	First population*		Relative abundance (%)	Second population*	Relative abundance (%)	Third population*	Relative abundance (%)	
84			M	*Corynebacterium.1*		82.2	*Staphylococcus*	3.9	*Propionibacterium*	2.9	
81			F	*Corynebacterium.1*		75.3	*Streptococcus*	5.6	*Neisseriaceae_D.5..Neisseria*	1.8	
10			F	*Corynebacterium.1*		64.0	*Staphylococcus*	18.1	*Rothia*	2.4	
40			M	*Corynebacterium.1*		54.7	*Staphylococcus*	30.2	*Propionibacterium*	6.0	
22			F	*Corynebacterium.1*		52.3	*Neisseriaceae_D.5..uncultured*	35.1	*Propionibacterium*	7.4	
43			F	*Corynebacterium.1*		35.7	*Staphylococcus*	23.0	*Propionibacterium*	17.5	
40			M	*Corynebacterium.1*		33.5	*Propionibacterium*	32.7	*Staphylococcus*	26.3	
43			F	*Staphylococcus*		73.4	*Propionibacterium*	6.2	*Lactobacillus*	5.8	
27			M	*Staphylococcus*		54.1	*Propionibacterium*	17.2	*Lawsonella*	13.5	

*Bacterial genera were ranked from those with high proportions.

**Table 1B:** Predominant genera in Stevens–Johnson patients.

Group	Age	Sex			First population*	Relative abundance (%)	Second population*	Relative abundance (%)	Third population*	Relative abundance (%)	Result of bacterial culture
1	51	M			*Corynebacterium.1*	99.8	*Escherichia.Shigella*	0.1	*Staphylococcus*	0.0	Not detected
	66	F			*Corynebacterium.1*	99.5	*Escherichia.Shigella*	0.2	*Propionibacterium*	0.2	Not detected
	30	M			*Corynebacterium.1*	99.4	*Propionibacterium*	0.1	*Escherichia.Shigella*	0.1	Not detected
	40	M			*Corynebacterium.1*	99.3	*Propionibacterium*	0.1	*Lawsonella*	0.1	*Serratia marcescens*
	69	F			*Corynebacterium.1*	99.2	*Escherichia.Shigella*	0.3	*Propionibacterium*	0.1	*Staphylococcus epidermidis*
	34	M			*Corynebacterium.1*	99.2	*Escherichia.Shigella*	0.3	*Propionibacterium*	0.1	Not detected
	58	M			*Corynebacterium.1*	98.9	*Neisseriaceae_D.5.uncultured*	0.9	*Staphylococcus*	0.1	*Staphylococcus epidermidis*
											*Corynebacterium amycolatum*
	80	F			*Corynebacterium.1*	98.4	*Neisseriaceae_D.5.uncultured*	0.8	*Acinetobacter*	0.2	*Staphylococcus species*
											*(coagulase negative)*
	60	F			*Corynebacterium.1*	98.2	*Escherichia.Shigella*	0.6	*Propionibacterium*	0.2	*Corynebacterium amycolatum*
	56	F			*Corynebacterium.1*	98.0	*Blastocatella*	0.5	*Escherichia.Shigella*	0.4	*Streptococcus oralis*
	38	F			*Corynebacterium.1*	97.8	*Staphylococcus*	1.9	*Escherichia.Shigella*	0.1	*Staphylococcus epidermidis*
	17	M			*Corynebacterium.1*	97.6	*Propionibacterium*	1.1	*Massilia*	0.5	Not detected
	17	M			*Corynebacterium.1*	96.7	*Neisseriaceae_D.5.uncultured*	1.8	*Escherichia.Shigella*	0.4	*Staphylococcus aureus*
											*Staphylococcus epidermidis*
	69	F			*Corynebacterium.1*	95.1	*Staphylococcus*	0.8	*Propionibacterium*	0.8	*Staphylococcus aureus*
											*Bacillius subtilis*
	62	F			*Corynebacterium.1*	93.2	*Roseomonas*	3.7	*Escherichia.Shigella*	1.2	Not detected
	26	M			*Corynebacterium.1*	92.7	*Propionibacterium*	2.9	*Escherichia.Shigella*	1.1	*Corynebacterium amycolatum*
	63	F			*Corynebacterium.1*	91.6	*Propionibacterium*	2.8	*Staphylococcus*	1.5	*Staphylococcus haemolyticus*
											*Corynebacterium amycolatum*
	68	F			*Corynebacterium.1*	89.8	*Finegoldia*	2.9	*Actinomyces*	2.4	*Corynebacterium amycolatum*
	60	F			*Corynebacterium.1*	87.6	*Escherichia.Shigella*	3.3	*Massilia*	1.8	Not detected
	26	M			*Corynebacterium.1*	81.1	*Propionibacterium*	15.6	*Staphylococcus*	0.8	*Staphylococcus aureus*
	55	F			*Corynebacterium.1*	79.4	*Streptococcus*	4.5	*Propionibacterium*	4.1	*Staphylococcus epidermidis*
											*Streptococcus agalactiae (GBS)*
	73	M			*Corynebacterium.1*	65.4	*Propionibacterium*	5.5	*Williamsia*	4.6	*Pseudomonas aeruginosa*
											*Staphylococcus haemolyticus*
											*Corynebacterium amycolatum*
	46	M			*Corynebacterium.1*	54.7	*Propionibacterium*	15.6	*Neisseriaceae_D.5.uncultured*	7.9	Not detected
	65	F			*Empedobacter*	52.1	*Corynebacterium.1*	47.2	*Streptococcus*	0.2	*Nonfermenting Gram Negative bacilli*
											*Corynebacterium amycolatum*
2	78	F			*Neisseriaceae_D.5.uncultured*	98.1	*Corynebacterium.1*	0.5	*Chryseobacterium*	0.4	*Leuconostoc species*
	50	F			*Neisseriaceae_D.5.uncultured*	92.5	*Corynebacterium.1*	2.1	*Lawsonella*	1.3	*Staphylococcus epidermidis*
	42	F			*Neisseriaceae_D.5.uncultured*	83.8	*Staphylococcus*	11.3	*Streptococcus*	3.1	Not detected
3	54	F			*Staphylococcus*	99.2	*Streptococcus*	0.3	*Corynebacterium.1*	0.3	*Streptococcus mitis*
											*Streptococcus oralis*
											*Staphylococcus epidermidis*
	38	M			*Staphylococcus*	95.2	*Corynebacterium.1*	2.4	*Streptococcus*	0.4	*Pseudomonas aeruginosa*
	51	F			*Staphylococcus*	84.7	*Corynebacterium.1*	11.8	*Escherichia.Shigella*	2.0	*Staphylococcus aureus*
4	81	F			*Escherichia.Shigella*	22.5	*Massilia*	17.6	*Staphylococcus*	8.4	*Staphylococcus haemolyticus*
	69	M			*Fusobacterium*	84.5	*Streptococcus*	7.1	*Prevotella.2*	4.5	Not examined
	37	F			*Massilia*	20.1	*Corynebacterium.1*	10.8	*Methylobacterium*	6.7	*Bacillus species*
	43	M			*Propionibacterium*	62.8	*Lawsonella*	13.0	*Solanum.melongena.eggplant.*	8.0	Not detected
	55	M			*Propionibacterium*	37.8	*Lawsonella*	31.7	*Staphylococcus*	13.0	*Staphylococcus caprae*
	48	F			*Serratia*	66.0	*Corynebacterium.1*	24.7	*Propionibacterium*	2.7	*Serratia marcescens*
	56	F			*Streptococcus*	96.8	*Corynebacterium.1*	2.2	*Aggregatibacter*	0.2	*Streptococcus pneumoniae*
											*Pseudomonas aeruginosa*

*Bacterial genera were ranked from those with high proportions.

### Altered Ocular Bacterial Composition in SJS/TEN Patients With SOC

**Figure 2:**
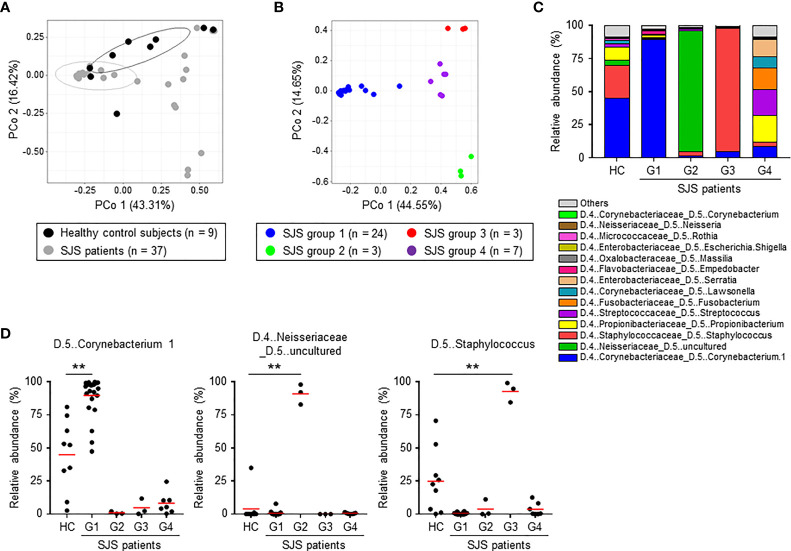
Composition of the ocular microbiome in healthy control subjects and SJS/TEN patients with SOC. **(A)** Principal coordinate analysis (PCoA) based on Bray–Curtis distance at the genus level was conducted to compare the composition of the ocular microbiome between healthy control subjects and SJS patients. **(B)** Classification of SJS patients into four groups based on the composition of the ocular microbiome at the genus level by PCoA. **(C)** Relative abundances (%) of the genera comprising the ocular microbiome of HC subjects and SJS patients. **(D)** Relative abundances (%) of the genera *Corynebacterium 1*, *Neisseriaceae uncultured*, and *Staphylococcus* in the ocular microbiome of HC subjects and SJS patients. Statistical significance was evaluated by one-way ANOVA; **p < 0.01. HC, Healthy controls (n = 9); G1, SJS group 1 (n = 24); G2, SJS group 2 (n = 3); G3, SJS group 3 (n = 3); G4, SJS group 4 (n = 7). SJS, SJS/TEN patients with SOC.

To clarify the composition of the ocular microbiome in SJS/TEN patients with SOC, we performed a principal coordinate analysis using Bray–Curtis distance at the genus level. We found that the composition of the ocular microbiome differed markedly between the HC subjects and SJS/TEN patients with SOC (**Figure 2A**). In addition, SJS/TEN patients with SOC could be divided into four groups based on the composition of their ocular microbiome (**Figures 2B**, **S1**). Group 1 was characterized by significant enrichment of species in genus *Corynebacterium 1* compared with that in the HC subjects and the other SJS/TEN patients with SOC groups (**Figures 2C, D**). Groups 2 and 3 were characterized by significant enrichment of species in genera *Neisseriaceae uncultured* and *Staphylococcus*, respectively. Group 4 was characterized by significant enrichment of species in several bacterial genera, including *Propionibacterium*, *Streptococcus*, *Fusobacterium*, *Lawsonella*, and *Serratia* (**Figure 2C**).

### Species-Level Diversity of *Corynebacterium 1* in SJS/TEN Patients With SOC

**Figure 3:**
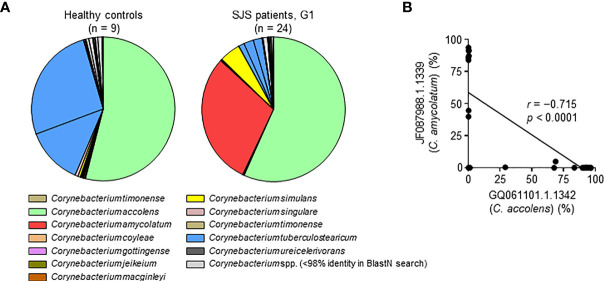
Species-level characteristics of genus *Corynebacterium 1* in healthy control subjects and SJS/TEN patients with SOC group-1 patients. **(A)** Results of a BlastN search of the 35 sequences of operational taxonomic units (OTUs) identified in genus *Corynebacterium 1*. The pie graph shows the mean percentage of each OTU and is color-coded according to the 12 *Corynebacterium* species estimated from the OTU sequences as shown in **Table S2**. **(B)** An exclusive relationship was found between the predominant *Corynebacterium 1* species *C. accolens* (OTU GQ061101.1.1342) and *C. amycolatum* (OTU JF087988.1.1339) among SJS/TEN patients with SOC group-1 (G1). *r*, Spearman’s correlation coefficient; *p*, *p*-value; SJS, SJS/TEN patients with SOC.

We next analyzed the ocular microbiome at the OTU level, focusing on the genera that were enriched in SJS/TEN patients with SOC. Thirty-five OTUs in genus *Corynebacterium 1* were detected (**Table S2**). Among these, OTU #GQ061101.1.1342 was found in both HC subjects and SJS/TEN patients with SOC group-1 patients and was identified as *C. accolens* by BlastN analysis using representative OTU sequences (**Figures 3A**, **S2** and **Table S2**). OTU #GQ006332.1.1341 and #FJ892757.1.1348, which were both identified as *C. tuberculostearicum*, were found in HC subjects but not in SJS/TEN patients with SOC group-1 patients. OTU #JF087988.1.1339, identified as *C. amycolatum*, was found in SJS group-1 patients (**Figures 3A**, **S2** and **Table S2**). Thus, genus two species from *Corynebacterium 1* (i.e., *C. accolens* and *C*. *amycolatum*) were found at high relative abundance in SJS/TEN patients with SOC. However, no SJS/TEN patients with SOC showed a high abundance of both *C. accolens* and *C. amycolatum*; some patients had a high abundance of *C. accolens* and a low abundance of *C. amycolatum* and *vice versa* (**Figure 3B**). This suggests that although genus *Corynebacterium 1* has species-level diversity in SJS/TEN patients with SOC, only a single *Corynebacterium 1* species tends to be dominant in a given SJS/TEN patients with SOC.

### Dominant Species From Genera *Neisseriaceae uncultured* and *Staphylococcus* in SJS/TEN Patients With SOC

Five and 10 OTUs were detected in genera *Neisseriaceae uncultured* and *Staphylococcus*, respectively (**Table S2**). In *Neisseriaceae uncultured*, OTU #JF224063.1.1380 was found at high abundance in HC subjects and SJS/TEN patients with SOC group-2 patients (**Figure S3** and **Table S2**). This representative OTU sequence was not matched to any sequence in the “rRNA_typestrains/16S_ribosomal_RNA” database (**Table S2**), suggesting the presence of an undefined bacterial strain in the ocular microbiome of SJS/TEN patients with SOC. In *Staphylococcus*, OTU #GBKB01000906.322.1853 was found at high abundance in HC subjects and SJS/TEN patients with SOC group-3 patients and was identified as *S. aureus* (**Figure S3** and **Table S2**). Thus, common bacterial OTUs in *Neisseriaceae uncultured* and *Staphylococcus* genera were detected in both HC subjects and SJS/TEN patients with SOC, respectively.

### Composition of the Ocular Microbiome in Both Eyes and Over Time

We next examined the differences in the ocular microbiome between the right and left eye of each SJS/TEN patients with SOC and the changes in the microbiome over time. We found no significant differences between the eyes with respect to the relative abundance of species in genera *Corynebacterium 1*, *Neisseriaceae uncultured*, and *Staphylococcus* (**Figure S4A**). Similarly, no significant changes in the relative abundance of species in genera *Corynebacterium 1*, *Neisseriaceae uncultured*, and *Staphylococcus* were observed at several months follow-up, although one patient did tend to show increased relative abundance for species in genus *Corynebacterium 1* and decreased relative abundance for species in genera *Neisseriaceae uncultured* and *Staphylococcus* (**Figure S4B**), and the patients did not show any significant changes of disease manifestation during the two time points of sample collection.

### Detection of *Corynebacterium*, *Staphylococcus*, and *Neisseriaceae* in SJS/TEN Patients With SOC by Bacterial Culture Test

Finally, to validate our 16S rRNA-based genetic analysis, we performed bacterial culture test used widely in clinical practice in 36 SJS/TEN patients with SOC. Potential bacterial pathogens such as *Staphylococcus*, *Corynebacterium*, *Streptococcus*, *Serratia*, *Pseudomonas*, *Leuconostoc*, *Bacillus* were isolated from 26 patients but not from 10 patients (**Table 1B**). *Staphylococcus* was most frequently detected in 15 of 36 patients (41.7%), followed by *Corynebacterium* in 7 of 36 patients (19.4%), and *Neisseriaceae* was not (**Table 1B**). Thus, the major bacterial species detected by the culture and sequencing methods are almost the same, demonstrating the validity of our 16S rRNA-based genetic analysis. In addition, these results revealed differences by methods in the frequency of detection and the culture method may fail to detect the presence of non-culturable bacteria such as *Neisseriaceae*.

## Discussion

Here, we characterized the ocular microbiome in Japanese HC subjects and SJS/TEN patients with SOC. A previous study conducted in Finland in healthy subjects showed high abundances of species in genera *Pseudomonas* (20%), *Propionibacterium* (20%), *Bradyrhizobium* (16%), *Corynebacterium* (15%), and *Acinetobacter* (12%) in the ocular microbiome (Dong et al., 2011). Another group has reported that species in genera *Staphylococcus* and *Propionibacterium* are abundant in the ocular microbiome in healthy Chinese subjects (Deng et al., 2020). Adding to this previous knowledge, we found that the ocular microbiome in Japanese healthy subjects was characterized by enrichment of species in genera *Corynebacterium 1* and *Staphylococcus*. Although, further studies with increased number of healthy individuals are needed, this information implicated a possibility that the ocular microbiome in healthy individuals might differ by geographic location.

When we examined the ocular microbiome in SJS/TEN patients with SOC, although it may be due to the fact that the patients are receiving antibiotics, we found that its composition differed compared with that in HC subjects. In addition, we were able to divide the SJS/TEN patients with SOC into four groups by the predominant genus or genera in the ocular microbiome: *Corynebacterium 1* (group 1); *Neisseriaceae uncultured* (group 2); *Staphylococcus* (group 3); or *Propionibacterium*, *Streptococcus*, *Fusobacterium*, *Lawsonella*, and *Serratia* (group 4). We found that there are multiple patterns of commensal bacteria on the ocular surface of SJS/TEN with SOC and also all groups included the patients with similarly varying severity of ocular sequelae, from mild to severe, suggesting that ocular microbiota might not correlate with severity of ocular sequelae. Therefore, it would be important to examine other eye conditions related to the severity. Accumulating evidence from studies on intestinal bacteria have shown that specific bacteria induced specific immune responses and consequently controlled inflammatory state (Garrett, 2020), raising a possibility that differences in bacterial compositions regulate inflammation in the eye. Alternatively, it is known that host immunity regulates the bacterial composition. Therefore, it remains unclear currently whether microorganisms are involved in the pathogenesis process or are a consequence of disease. In addition, at present, treatment methods according to the ocular microbiota are difficult to determine, which is a subject of our future study.

Previously, we postulated that a balance exists between an individual’s innate immunity at the ocular surface and the pathogenicity of bacteria; that is, when the host innate immune system is functioning normally, commensal bacteria are in a symbiotic relationship with the host, whereas if the host’s innate immune system is not functioning normally, commensal bacteria can become pathogenic (Ueta et al., 2007a). Our present data show that the ocular microbiome in SJS/TEN patients with SOC had reduced diversity compared with that in HC subjects, which may indicate that the innate immune system is not functioning normally in SJS/TEN patients with SOC, leading to commensal bacteria becoming pathogenic.

Moreover, in SJS/TEN with SOC patients, an increase and/or alteration in ocular microbiota, for example when their discharge increase, could exacerbate their ocular surface inflammation. Therefore, the control of ocular microbiota by reducing the amounts of bacteria might be important for reducing their ocular surface inflammation. Therefore, we believe that it makes sense to examine ocular microbiota to reduce the ocular inflammation.

The 16S rRNA-based genetic analysis revealed that the ocular microbiome of 24 of 37 (64.9%) SJS/TEN patients with SOC was characterized by enrichment of species in genera *Corynebacterium 1* (group 1). This was surprising because species in genus *Staphylococcus* were detected by bacterial culture test in 15 of 36 SJS/TEN patients with SOC (41.7%) (one patient was not examined), whereas species in genus *Corynebacterium* were detected in only 7 of 36 SJS/TEN patients with SOC (19.4%). Also, *Staphylococcus* species were detected by bacterial culture test in all SJS/TEN patients with SOC showing enrichment of species in genera *Staphylococcus* (group 3), whereas *Corynebacterium* species were detected in only 7 of 24 SJS/TEN patients with SOC (29.2%) showing enrichment of species in genera *Corynebacterium 1* (group 1). Culture-based methods are known to be influenced by sample collection and culture conditions and that only a limited range of bacterial species can be grown in culture (Zegans and Van Gelder, 2014; Kugadas and Gadjeva, 2016). Therefore, these results suggest not only that *Staphylococcus* species are more easily detected than *Corynebacterium* species by bacterial culture, but also that 16S rRNA-based genetic analysis represents an improved and significantly higher-resolution method for the detection of microbial species.

Within genus *Corynebacterium*, we found that *C. accolens* and *C. amycolatum* were enriched in SJS/TEN patients with SOC group-1 patients, whereas *C. accolens* and *C. tuberculostearicum* were enriched in HC subjects. Thus, *C. amycolatum* was enriched in SJS/TEN patients with SOC group-1 patients, but not in HC subjects. In addition, genus *Corynebacterium 1* showed species-level diversity among SJS/TEN patients with SOC, but only a single species from the genera was enriched in a given patient. In contrast, greater species-level diversity was observed in HC subjects. *Corynebacterium amycolatum*, but not *C. accolens*, was detected by bacterial culture test in 7 of 24 (29.2%) SJS/TEN patients with SOC group-3 patients. Because *C. accolens* might be difficult to detect with current bacterial culture test technology, our results suggest improved culture methods or novel detection systems are needed.

*Corynebacterium*, except for diphtheria-causing species *C. diphtheriae*, are dominant members of the skin microbiota and associated with opportunistic infection and the development of inflammatory skin diseases (Byrd et al., 2018; Olejniczak-Staruch et al., 2021). For example, under steady-state conditions in mice, *C. accolens* does not cause inflammation; however, when this steady state is disrupted, *C. accolens* promotes inflammation *via* a bacterial cell wall component in an interleukin 23–dependent manner (Ridaura et al., 2018). Similarly, on the ocular surface, *Corynebacterium* species are commonly found in both healthy individuals and individuals with inflammatory ophthalmic conditions such as keratitis and conjunctivitis (Aoki et al., 2021). Indeed, *C. accolens* and *C. amycolatum* have been isolated from ocular samples obtained from patients with bacterial conjunctivitis and corneal ulcer, respectively (Eguchi et al., 2008; Sugumaran et al., 2020). *Staphylococcus* species such as *S. epidermidis* and *S. aureus* are well-known skin colonizers that are also associated with inflammatory skin diseases such as atopic dermatitis in humans (Kong et al., 2012; Nakamizo et al., 2015; Byrd et al., 2018). In addition, these species have been shown in a murine atopic dermatitis model to be factors that drive inflammation (Kobayashi et al., 2015). Furthermore, *Staphylococcus* species are frequently isolated from ocular samples and are recognized as causative factors of inflammatory ophthalmic diseases such as keratitis and conjunctivitis (Somerville et al., 2020). Thus, in ocular tissue and the surrounding skin, *Corynebacterium* and *Staphylococcus* species exist as commensals that have the potential to induce inflammation. *Neisseriaceae* species are also associated with skin inflammation and ocular infection (Teweldemedhin et al., 2017; Hsu et al., 2020), although there is a lack of genomic database information.

The present findings suggest that species in these genera could be pathogens related to SJS/TEN patients with SOC. In addition, it would be important to compare with other ocular surface disorders, and the exploration of factors among each subgroup is required for further studies.

Mucosae usually do not respond to commensal bacteria; however, the ocular surface in SJS/TEN patients with SOC with abnormal mucosal innate immunity might respond to commensal bacteria (Ueta and Kinoshita, 2012). Previously, we have suggested that abnormal mucosal innate immunity might contribute to ocular surface inflammation in SJS/TEN patients with SOC (Ueta and Kinoshita, 2012).

Collectively, the present findings indicate that the composition of the ocular microbiome differs between healthy individuals and SJS/TEN patients with SOC. In addition, the ocular microbiome was found to differ among the SJS/TEN patients with SOC, with four different genera-enrichment patterns observed. The changes in the ocular microbiome of SJS/TEN patients with SOC may be caused by abnormal host mucosal innate immunity and could be associated with the ocular surface inflammation often observed in SJS/TEN patients with SOC.

## Data Availability Statement

The datasets presented in this study can be found in online repositories. The names of the repository/repositories and accession number(s) can be found below: https://www.ddbj.nig.ac.jp/, PRJNA737344.

## Ethics Statement

The studies involving human participants were reviewed and approved by the National Institutes of Biomedical Innovation, Health, and Nutrition Kyoto Prefectural University of Medicine. Written informed consent to participate in this study was provided by the participants’ legal guardian/next of kin.

## Author Contributions

MU, KH, and JK designed the study and wrote the manuscript. MU, CS, and SK collected the human samples. MU, CS, SK, and KH performed the microbiome analysis. JP and KM performed the bioinformatics analysis. All authors contributed to the article and approved the submitted version.

## Funding

This work was supported by the Ministry of Education, Culture, Sports, Science and Technology of Japan and Japan Society for the Promotion of Science KAKENHI (grant numbers 18K17997 and 19K08955 to KH; 18H02674, 20H05697, 20K08534, 20K11560, 18H02150, and 17H04134 to JK; 19H03809 to MU and CS; 21K09749 to MU; and 21K15267 to JP); the Japan Agency for Medical Research and Development (JP20ek0410062h0002, 20fk0108145h0001, JP20ak0101068h0004, and JP20gm1010006h004 to JK); the Ministry of Health and Welfare of Japan and Public/Private R&D Investment Strategic Expansion PrograM: PRISM (grant number 20AC5004 to JK); the Ministry of Health, Labour and Welfare of Japan (grant number JP19KA3001 to KH); the Cross-ministerial Strategic Innovation Promotion Program: SIP (grant number 18087292 to JK); and grants from the Joint Research Project of the Institute of Medical Science, the University of Tokyo (to JK), the Ono Medical Research Foundation (to JK); and the Canon Foundation (to JK).

## Conflict of Interest

The authors declare that the research was conducted in the absence of any commercial or financial relationships that could be construed as a potential conflict of interest.

## Publisher’s Note

All claims expressed in this article are solely those of the authors and do not necessarily represent those of their affiliated organizations, or those of the publisher, the editors and the reviewers. Any product that may be evaluated in this article, or claim that may be made by its manufacturer, is not guaranteed or endorsed by the publisher.

## Supplementary Material

Supplementary Figure 1 Classification of SJS patients into four groups based on the composition of the ocular microbiome at the genus level by hierarchical clustering analysis. SJS, SJS/TEN patients with SOC.

Supplementary Figure 2 Relative abundances (%) of species of genus *Corynebacterium 1* in healthy control (HC) subjects and SJS/TEN patients with SOC group-1. SJS, SJS/TEN patients with SOC.

Supplementary Figure 3 Species-level characteristics of genera *Neisseriaceae uncultured* and *Staphylococcus* in healthy control subjects and SJS/TEN patients with SOC group-2 and -3, as determined by BlastN search using the identified representative operational taxonomic unit sequences. SJS, SJS/TEN patients with SOC.

Supplementary Figure 4 Composition of the ocular microbiome in SJS/TEN patients with SOC did not differ by right *vs.* left eye or time course. **(A)** Relative abundances (%) of genera *Corynebacterium 1*, *Neisseriaceae uncultured*, and *Staphylococcus* in the right and left eyes of SJS/TEN patients with SOC. **(B)** Relative abundances (%) of genera *Corynebacterium 1*, *Neisseriaceae uncultured*, and *Staphylococcus* in SJS/TEN patients with SOC over several months. Because samples were taken at the patient’s visit to hospital, the time periods vary by sample with seven samples at 6–7 months interval and two samples at 1–2 months interval. Statistical significance was evaluated by using the Wilcoxon rank sum test; n.s., not significant; SJS, SJS/TEN patients with SOC. For ethical and experimental reasons, the samples shown in this figure do not overlap completely with those shown in other figures.
