# Supplementary figures and images for: Categorization of the Ocular Microbiome in Japanese Stevens–Johnson Syndrome Patients With Severe Ocular Complications
Source: Front Cell Infect Microbiol. 2021 Nov 19;11:741654. doi: 10.3389/fcimb.2021.741654 (PMC8640524; doi:10.3389/fcimb.2021.741654)

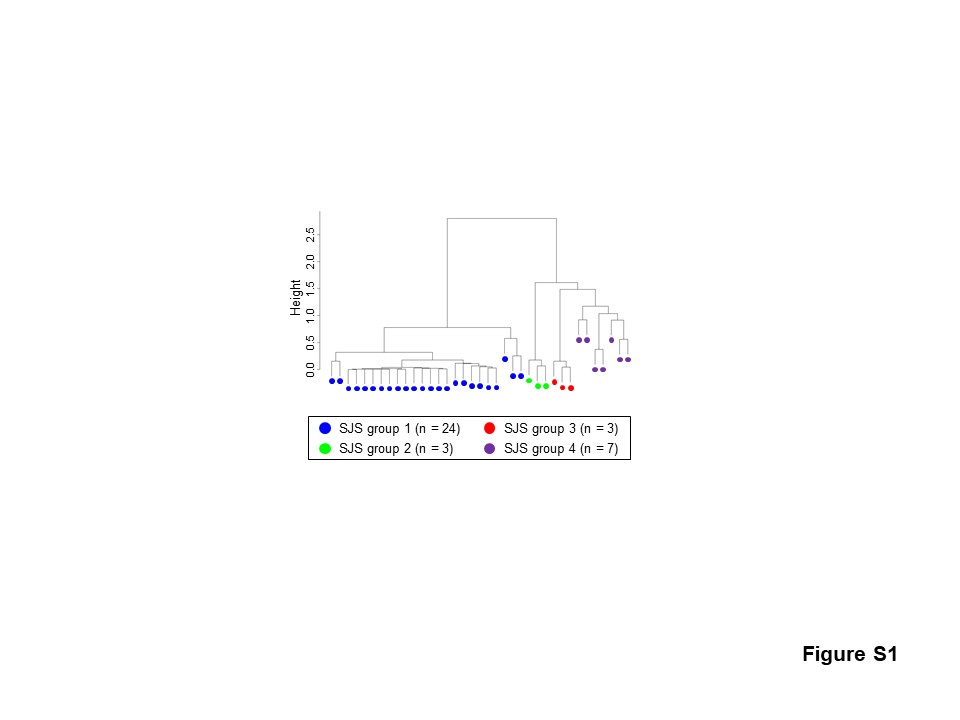

Supplement: Supplementary Figure 1 — Classification of SJS patients into four groups based on the composition of the ocular microbiome at the genus level by hierarchical clustering analysis. SJS, SJS/TEN patients with SOC. [file Image_1.tif]

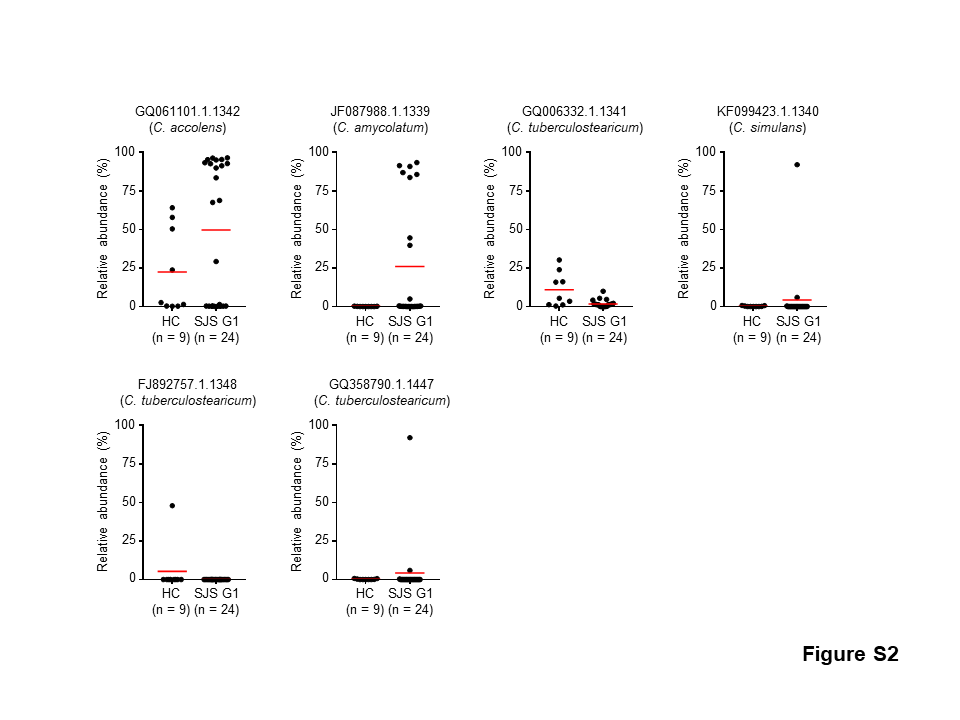

Supplement: Supplementary Figure 2 — Relative abundances (%) of species of genus Corynebacterium 1 in healthy control (HC) subjects and SJS/TEN patients with SOC group-1. SJS, SJS/TEN patients with SOC. [file Image_2.tif]

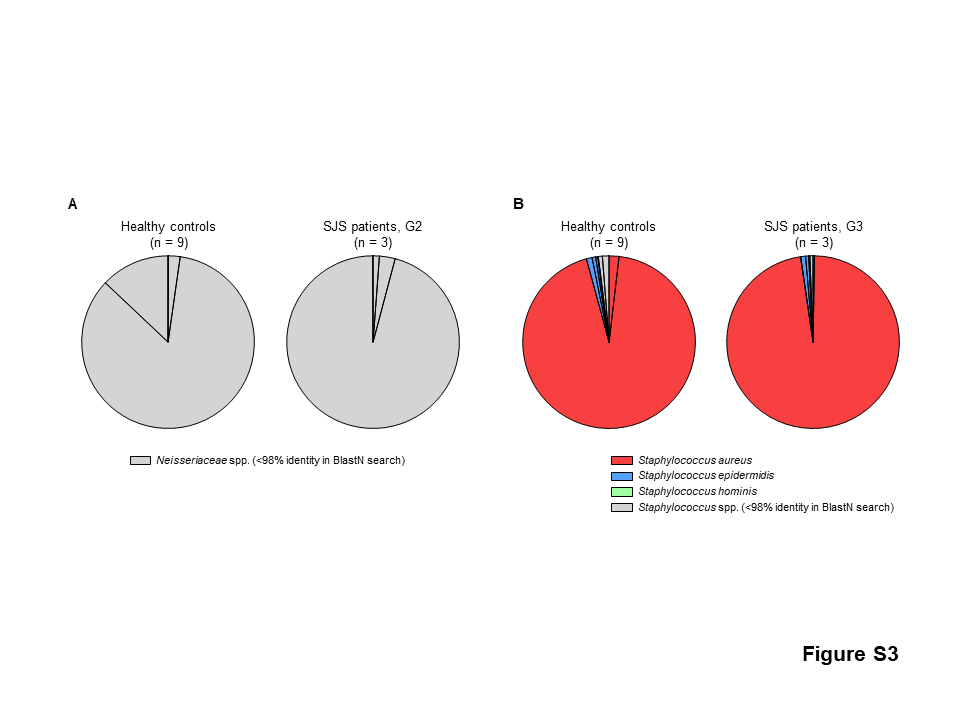

Supplement: Supplementary Figure 3 — Species-level characteristics of genera Neisseriaceae uncultured and Staphylococcus in healthy control subjects and SJS/TEN patients with SOC group-2 and -3, as determined by BlastN search using the identified representative operational taxonomic unit sequences. SJS, SJS/TEN patients with SOC. [file Image_3.tif]

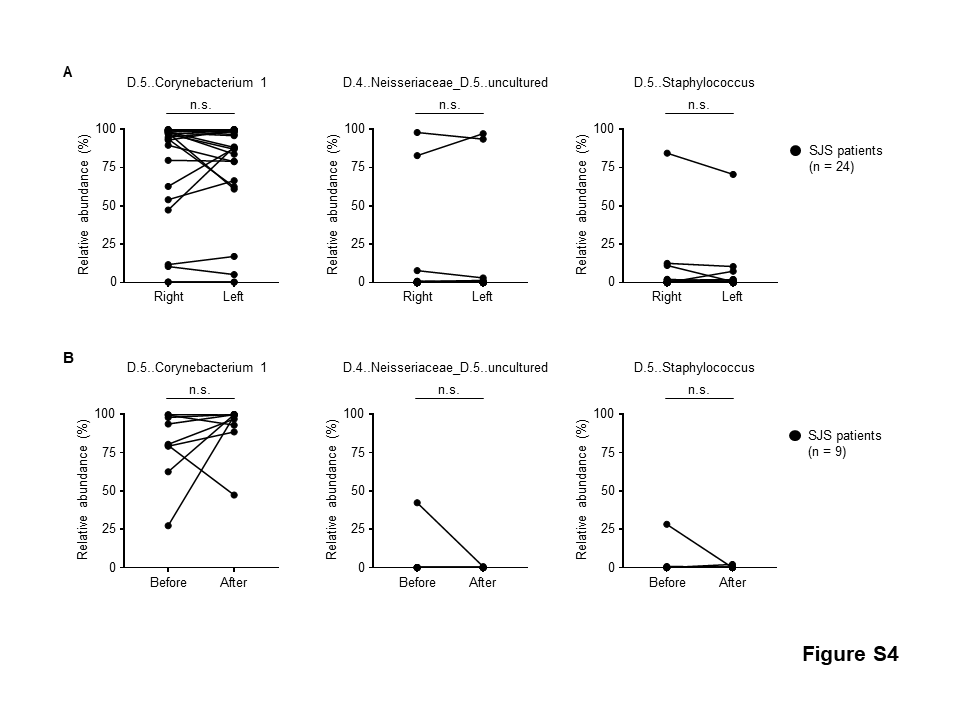

Supplement: Supplementary Figure 4 — Composition of the ocular microbiome in SJS/TEN patients with SOC did not differ by right vs. left eye or time course. (A) Relative abundances (%) of genera Corynebacterium 1, Neisseriaceae uncultured, and Staphylococcus in the right and left eyes of SJS/TEN patients with SOC. (B) Relative abundances (%) of genera Corynebacterium 1, Neisseriaceae uncultured, and Staphylococcus in SJS/TEN patients with SOC over several months. Because samples were taken at the patient’s visit to hospital, the time periods vary by sample with seven samples at 6–7 months interval and two samples at 1–2 months interval. Statistical significance was evaluated by using the Wilcoxon rank sum test; n.s., not significant; SJS, SJS/TEN patients with SOC. For ethical and experimental reasons, the samples shown in this figure do not overlap completely with those shown in other figures. [file Image_4.tif]
